# Nurses’ justifications for morally courageous acts in ethical conflicts: A narrative inquiry

**DOI:** 10.1177/09697330241284357

**Published:** 2024-09-26

**Authors:** Elina Pajakoski, Helena Leino-Kilpi, Minna Stolt, Anto Čartolovni, Riitta Suhonen

**Affiliations:** 8058 University of Turku; 324714 Catholic University of Croatia; 8058 University of Turku

**Keywords:** Ethical conflict, holistic analysis, justification, moral courage, narrative inquiry, registered nurse

## Abstract

**Background:** Moral courage is defined as the courage to act in ethical conflicts based on individual or professional values despite the personal risks involved. Nurses justify their decisions to act morally courageously as part of their ethical decision-making.

**Objective:** To describe registered nurses’ justifications for acting morally courageously, or not, in ethical conflicts where they needed moral courage.

**Research design:** A narrative inquiry with a holistic content approach was used. Individual, in-depth interviews were conducted in January–February 2023. The data were analysed using holistic content analysis.

**Participants and research context:** Fourteen registered nurses with experience in situations where they needed moral courage participated. The nurses came from the somatic, palliative, mental health, and substance abuse care fields in Finland.

**Ethical considerations:** Good scientific practice was followed. Ethical approval was obtained before data collection from the university’s ethics committee.

**Findings:** The nurses needed moral courage in ethical conflicts with patients present and between professionals. Individual responsibility, professional ethics, and emotions were identified as bases of nurses’ justifications for morally courageous acts. The justifications for acting morally courageously, or not, had individual, contextual, and organisational perspectives. Morally courageous acts included starting a discussion about the conflict with other professionals and reporting the situation in writing within one’s organisation.

**Discussion and conclusions:** The identified bases and perspectives of justifications illustrate the complexity of nurses’ decision-making in ethical conflicts, either leading to morally courageous acts or not. These results can inform nursing practice and research in developing processes to strengthen nurses’ moral courage and examining relationships between moral courage and other concepts, such as moral resilience.

## Introduction

Moral courage is a valued personal characteristic and a virtue, with which nurses can promote quality of care and advocate for patients.^1,2^ It is defined as the courage to act based on individual or professional values in an ethical conflict, despite personal risks from the act.^1,2^ Nurses encounter ethical conflicts when they aim to fulfil their professional duty to do good for their patients^3,4^ and respect human dignity and autonomy.^3^ Ethical conflicts can stem from differing views of professionals and patients or their next of kin or challenges in collaboration between professionals.^5^ Also, organisational issues, such as the nurse shortage or the hierarchy in decision-making processes, can involve ethical conflicts.^6^ According to the definition of moral courage,^1^ ethical conflict and personal risk are always present when a nurse acts morally courageously.

Research on nurses’ moral courage, including concept analyses,^1,2^ literature reviews,^7,8^ and empirical studies, is increasing. Empirical studies have been conducted from the perspectives of nurses,^9–16^ nurse students,^17–20^ and nurse managers.^21^ In empirical research, moral courage is described as a part of nurses’ ethical competence, highlighting its relation to professional nursing ethics.^1,22^ Sometimes nurses even go beyond their ethical responsibilities with morally courageous acts.^23^ Associated factors include earlier life and work experience,^18^ knowledge about and skills in ethics,^9^ and ethical climate in organisations.^11^

Despite increases, research focusing on nurses’ justifications for their morally courageous acts is still scarce. A nurse decides whether to act morally courageously based on ethical reasoning, forming a link between moral courage and ethical decision-making.^24^ Ethical decision-making and acting in ethical conflicts can be based on nurses’ professional values and decision-making frameworks, including the ICN Code of Ethics for Nurses or the WHO Global Health Ethics Key Issues.^3,25^ Morally courageous acts can be justified individually, or with colleagues, co-workers, or patients.^26^ After establishing a justification, nurses act morally courageously despite possible negative consequences for themselves.^24^ Nurses often initiate discussions about ethical conflicts, acting morally courageously.^22,27^ Initiating a discussion or filing a note can be done inside the organisation, for example, with the closest manager. Also, nurses can inform someone outside the organisation, such as a trade union representative.^28^ This study focuses on nurses’ justifications for their morally courageous acts. Describing justifications promotes learning from nurses’ experiences, which can support decision-making and potentially offer tools that strengthen nurses’ moral courage.^29^

## Objective and research questions

The objective of this study was to illustrate registered nurses’ justifications for acting morally courageously or not in ethical conflicts where they need moral courage.

The following research questions were set:

1. In which ethical conflicts do nurses need moral courage?
2. What are nurses’ justifications for acting morally courageously, or not, in ethical conflicts?

## Methods

### Research design

In a narrative inquiry, Lieblich’s holistic content approach was used.^29^ This approach involves asking participants to talk about their experiences in an undisturbed narrative and analysing the transcribed narratives as a whole. Thus, the ‘narrative’ in this study refers to the contents of the participants’ narratives in their interviews. Each point of interest, according to the research questions, is followed throughout the transcription of each interview. The holistic content approach allows for the identification of ethical conflicts as context for morally courageous acts, and deep and broad meanings of the nurses’ justifications for whether to act morally courageously. The chosen approach also facilitates a meaningful and comprehensive presentation of the integrated findings from the participants’ narratives.^29^ This report follows the Consolidated Criteria for Reporting Qualitative Studies (COREQ).^30^

### Sampling and context

Purposive sampling was conducted from a large Finnish nursing-themed online discussion forum, hoitajat.net,^31^ to reach registered nurses with experience of ethical conflicts and the need for moral courage.^29^ The discussion forum was chosen because it is accessible to all nurses and has a dedicated section for invitations to participate in studies.^31^ The first author published an invitation for participation and information about the study as part of doctoral research with a link to a detailed information letter and a privacy notice.^32^ To be included, participants had to be registered nurses, who had identified ethical conflicts in their work and had needed moral courage to solve these conflicts, whether they acted morally courageously or not. The nurses themselves determined whether they had the required experience to participate. Participants could join by contacting the first author by phone or email. Moral courage was analysed in relation to ethical conflicts. Thus, the research context was the ethical conflicts the participants had encountered in professional nursing in Finland. This paper describes the complex ethical conflicts as part of the holistic illustration of nurses’ justifications to facilitate a deep and broad understanding of the justifications.

### Data collection

The participants’ age (years), work experience in healthcare (years), current and previous work roles, and fields of nursing were collected before the interviews with a short questionnaire either on paper or in a digital format, according to each participant’s wishes. Two pilot interviews were conducted to test the usefulness and clarity of the interview scheme. No changes to the interview scheme were made after the pilot.^30^ The first author conducted individual open interviews, to appreciate the uniqueness of each participant’s experience.^29^ The interviews took place between January and February 2023 either using the virtual meeting platform Zoom^33^ or face-to-face, according to each participant’s wishes. The interviews were recorded with each participant’s written consent. Only the interviewer and the participant were present in the interview. The interview started with a request for the participant to share experiences of ethical conflicts in which they would have needed moral courage but did not act morally courageously. Then, the interviewer asked the participant to talk about ethical conflicts in which they had needed moral courage and had acted morally courageously. Each participant told varying examples in their narrative. After the participants provided the initial narrative, the researcher asked probing questions when necessary.^29^ The interviewer would ask ‘What was the justification for acting or not acting morally courageously?’ to obtain more detailed information about the participant’s justification if the participant did not explain them initially. After each ∼25 to ∼90-min interview, the interviewer took notes to assist the data analysis.^34^ The notes comprised the participant’s tone of voice, body language, and the emotions that their non-verbal communication could have represented. Recruiting participants and conducting interviews continued until theoretical saturation was reached. This was assessed continuously throughout the data collection. When no new knowledge regarding ethical conflicts, justifications, or morally courageous acts was identified in the interviews, theoretical saturation was deemed to have been reached.^32^

### Data analysis

The interview data were analysed using holistic content analysis, following Lieblich’s holistic content approach.^29^ The field notes were used to revisit the atmosphere of each interview. This enhanced maintaining the holistic perspective during the analysis. Faithfully to the holistic content approach, each transcribed interview was handled as a whole instead of analysing it in pieces. The results of the analysis represent the researchers’ interpretations of the meanings of the data.^29^ The first author was responsible for the analysis, which was regularly discussed within the research team (E.P., H.L-K, and R.S).

First, each participant’s narrative from their transcribed interview was analysed separately: then, the findings from all participants’ interviews were merged. The steps of the analysis^29^ were: (1) Each text was read several times to gain a holistic idea of the text; (2) content in the narrative relevant to the study objective was identified: the ethical conflicts where nurses needed moral courage, the justifications for whether to act morally courageously, and what was the action taken; (3) areas of ethical conflicts and justifications were identified; (4) Steps 1–3 were repeated with each narrative; (5) nurses’ bases of justifications were identified from content that was identified repeatedly in the nurses’ justifications; (6) based on the similarities and differences in justifications for morally courageous acts, three perspectives of justifications were formed. The perspectives illustrate specific viewpoints for justifications in each ethical conflict, while the bases of justifications represent a broad general basis for nurses’ justifications in all ethical conflicts; (7) the ethical conflicts, relevant justification, and the morally courageous act were identified holistically, according to the bases and perspectives of justifications; (8) all nurses’ justifications and their perspectives for not acting morally courageously were identified and described together. The participants described their justifications for whether or not to act morally courageously with varying depth in their narratives. The identified bases and perspectives of justifications are the results of the analysis of the content of the initial narratives Supplementary Table S1.^29^

## Participants

Fourteen registered nurses participated. They had from seven to over thirty years of working experience in healthcare. The participants had worked in somatic, palliative, mental health and substance abuse care, inpatient hospital wards, outpatient clinics, and emergency services, with patients from all age groups. Additionally, some participants were currently working or had previously worked as staff nurses, nurse managers, or practical nurses.

### Ethical considerations

Research ethics were followed.^35^ Ethical approval was received from the Ethics Committee of University of Turku on 24 October 2022. This study deals with the moral activity of individuals; the topic is personal and can be perceived as sensitive. The participants were healthcare professionals, who had received information about the study and had given written informed consent before voluntary participation. Participants’ personal data were processed following the EU Data Protection Directive (679/2016).^18^

## Findings

First, the ethical conflicts described by the nurses in their narratives and their need for moral courage are presented. Second, the bases and perspectives of justifications for morally courageous acts are described. Finally, the justifications for not acting morally courageously are described.

### Ethical conflicts in which the nurses needed moral courage

The ethical conflicts that the nurses described in their narratives either involved the patient or their next of kin or were situations that arose between professionals. Areas of conflict involving a patient or next of kin were missed care, a threat to patient safety, truth and privacy, and respect for others. ‘So there the unit’s nurses are holding the old patient down forcefully with both hands… so then the I.V. is in, and the drip is on… and… and this patient screams very loud… this old patient… and… these nurses say that the patient has dementia and therefore does not get to decide themselves…’ (Participant 6).

The areas of conflict between professionals were equality regarding work tasks, collaboration, privacy, and respect for others. ‘The nurse leader… well… she started to bully me … she started to stalk me, she asked a couple of nurses… [they were] kind of her trusted nurses, to stalk me and tell her what I did’ (Participant 14) (Table 1).

**Table 1.** The areas of ethical conflicts in the study.

Area of conflict	Description of conflict
A patient or next of kin is present in the situation	
Missed care	Missed care in the unit because of an insufficient number of nurses
	Equity in getting the correct care at the correct time is not actualising in the unit due to the work processes not adjusting to the individual patient’s needs
	Missed care because of lack of competence in the unit
	The patient’s right to sufficient pain relief is not actualising
Threat to patient safety	The patient’s well-being is threatened
	Patient’s rights for good care are not actualising
	Patient safety is threatened
Truth and privacy	Patient’s privacy is violated
	Patient’s right to knowledge of their condition is not actualising
Respect	Other professional acts unprofessionally, even rude, towards a patient, missing respect and compassion
	The patient’s next of kin acts threateningly towards the nurse due to a disagreement regarding the patient’s care
Between professionals, patient(s) not present	
Equality in work tasks	Work tasks are divided unequally in the unit: some nurses must do more tasks
	Work tasks are unequally balanced between different care units due to insufficient competence in one unit
	Tasks unrelated to patient care are required from nurses, leading to the patients getting insufficient care
Collaboration	A tense atmosphere and challenges in communication between professional groups about the unfair balance of work tasks
	Communication and collaboration challenges between care units about the unfair balance of work tasks
Privacy	A nurse’s privacy regarding their health is violated
Respect	Nurse leaders and/or colleagues do not respect some colleagues, even bullying them
	Lack of an individual care plan when a physician and a nurse view the needs of the patient differently
	Older or more experienced nurses do not respect younger or newer ones

### Nurses’ justifications for morally courageous acts

The justifications described by the nurses in their narratives are presented from abstract to more concrete. A nurse’s professional identity and the aim of doing good for the patient were a common starting point for the justifications of all participants. Then, each nurse had a basis of justification: internal responsibility, professional ethics, or emotions. While the basis of justification was the foundation for justifying morally courageous acts in every ethical conflict, the perspective of justification was a more specific point of view of justifications, varying in each situation. In some cases, there was more than one justification. However, with the aim of presenting the justifications clearly, their separate bases and perspectives are described (Table 2).

**Table 2.** Bases and perspectives of justifications for morally courageous acts.

	Bases of justifications		
	Internal responsibility	Professional ethics	Emotions
Perspective of justification	Justifications for the morally courageous act ‘Acts morally courageously based on…’		
Individual	Own values	Moral responsibilities as a professional	The willingness to ensure others’ (professional or patient) or own well-being
	Own professional and moral competence	Responsibility to promote nurses being valued in the community	The willingness to do the right thing for the patient
	Own earlier experiences		Balancing between one’s own and the patient’s well-being
	Own conscience		The goal to ensure the patient’s autonomy is actualised
Contextual	Respect for the patient’s view	The situation continuing or repeating	The situation is harmful to the patient
	The severity of the situation	The responsibility to develop care processes to be more functioning for the patients	The emotions awakened in the situations (e.g. being angry about wrongdoing or wrongness)
Organisational	Support from others (manager and colleagues)	Another person telling or ordering to act	

### Bases of justifications

Internal responsibility as a basis of justification was related to the nurse’s values, competence, experiences, and conscience. ‘… something just… deep inside says that this is right, or this is wrong, and I always act according to what I feel is right’ (Participant 10). Professional ethics as a basis for justification was related to professional responsibilities, such as providing quality care with functioning processes. ‘… cos it’s my area of responsibility, so… of course, it’s that we have to battle a lot you know… on behalf of the patients’ (Participant 2). Finally, having emotions as a basis for justification was linked to the nurses’ willingness to make sure everyone was feeling well, willingness to do the right thing, and getting angry about the wrongness of a situation. They identified a violation of professional values, and, for example, got angry about it. Emotion was the driving force for acting morally courageously. ‘I mean, it was just the death of the patient then, the smaller things didn’t wake me up… and then I got angry’ (Participant 13) (Table 2).

### Perspectives of justifications

In addition to the bases of justifications, the nurses’ justifications in each ethical conflict had an individual, contextual, or organisational perspective. The individual justifications were related to the nurses themselves and their viewpoints, while the contextual justifications were related to the severity, timing, and other attributes of the situation or the surroundings. The organisational justifications were related to other professionals and the organisational matters, such as a well-being strategy in the organisation (Table 2).

### Holistic illustrations of the ethical conflicts, justifications, and morally courageous acts

**Figure 1.**
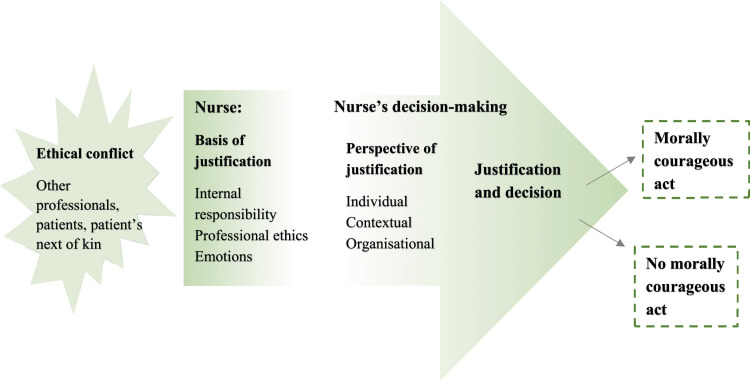
Nurse as a central actor in the decision-making process in ethical conflicts, leading to a morally courageous act.

Next, examples of ethical conflicts and related justifications for acting morally courageously as well as morally courageous acts are presented according to each basis and perspective of justification. After purposefully identifying their justification or justifications, the nurses decided to act morally courageously (Table 3, Figure 1).

**Table 3.** Ethical conflicts, justifications, and morally courageous acts.

	Bases of justifications		
	Internal responsibility	Professional ethics	Emotions
Individual justification			
Ethical conflict and justification	Missed care due to lack of competence in the unit. Nurses from other units had to help and support the unit, leaving tasks undone in their own units. Own earlier experiences about not being able to act morally courageously give strength to act courageously	In a care situation, a colleague was rude and disrespectful to the patient and handled them roughly. Knowing own obligations to promote good care and smooth care processes are justification to act	A colleague told untruths about a patient to the physician, for example, to get the right to restrict the patient. Also, the colleague diminished the patient’s wishes. The participant felt that the situation was wrong from the patient’s point of view. Respected the patient’s rights and was ready to act to support them
Authentic quote	‘Maybe the [earlier unsuccessful] experience… helped me, you know, to act. It was kind of my own motive, to be able to make some things better’ (Participant 7)	‘They are difficult… difficult [situations] to confront but everyone kind of silently knows about it. It’s not only that one time, but also a kind of continuous behaviour’ (Participant 1)	‘I’m there for the patients’ sake and it can’t go the way that… if a person comes in the care and they get more damage than help’ (Participant 9)
Morally courageous act	The morally courageous act was to discuss with their own team and afterwards with the head nurse. The first discussion did not change anything. After speaking to the head nurse, the matter was further discussed	The morally courageous act was to open discussion after first telling someone else and the other person inviting people into the discussion	The participant gave their own opinion in the discussion, defending the patient’s rights
Contextual justification			
Ethical conflict and justification	A severely injured patient was not getting all the examinations that were needed because of a (young) physician’s lack of competence. It was a high risk for the patient. The morally courageous act was based on the severity of the situation	In a care situation, a colleague was rude to the patient, spoke disrespectfully and handled them roughly, causing pain for the patient. Continuity of the situation as a justification: Acted later, when the same kind of situation happened again or continued	Practical nurses refused to work in a team with registered nurses, saying that they did not need a registered nurse. A patient died because of a lack of treatment. The justification for a morally courageous act was being angry because of the wrongness. The death of the patient led to angriness, which gave strength to act morally and courageously
Authentic quote	‘And why I did that is that I was deeply concerned about the patient’s condition… I mean, there was a high risk for further injuries’ (Participant 4)	‘So that’s probably the hardest part, how you talk in the situation. I Can say that I will eventually go and… and monitor the situation… and… yes, I do take it forward. But it’s hard’ (Participant 1)	‘ … the angriness was the driving force then, that this won’t end here… and when I got the ball rolling… then there was nothing that could top it… but you know, it was very difficult. But I just couldn’t cope with the wrongness’ (Participant 13)
Morally courageous act	The morally courageous act was to speak politely to the person involved	Did not speak to the person involved but pondered alone and later told someone else. Also, filed a written notice to the organisation	The act was to speak straight to the involved persons and send a letter to the head nurse and nurse manager in the unit. A big meeting was arranged. Later, the nurse wrote a letter to local politicians and professionals
Organisational justification			
Ethical conflict and justification	A palliative patient was suffering and death was approaching. The patient did not get the necessary care and medications in the unit. The justification for the act was that the nurse knew another physician would understand and support her and the patient’s rights	A nurse leader told the nurse’s private things and lies about the nurse publicly in the unit. The leader’s own manager supported the leader and claimed there was no proof of this. Justification was support from colleagues	-
Authentic quote	‘A very lovely physician, who… really thoughtfully likes to take care of these palliative care patients --- I have a feeling that I don’t have to explain why I want something for the patient, as our view of ethical care is similar’ (Participant 5)	‘… in the end, as a support, I’ve had my own group there… and there they are, you know, those good workmates from whom I get the help and support’ (Participant 12)	
Morally courageous act	The morally courageous act was to speak up, and when nothing changed, contact another person in the organisation	The morally courageous act was to tell a manager higher in the organisation about the situation and to human resources team

### Nurses’ justifications for not acting morally courageously

The nurses identified the ethical conflict and the need for moral courage even when they did not act morally courageously. Not acting courageously often left them morally distressed and feeling bad about the situation even after many years. Justifications for not acting morally courageously were similar between nurses and are thus described together (Table 4).

**Table 4.** All participants’ justifications for not acting morally courageously.

Justification	Ethical conflict
Individual	
Fear of being left alone	A coworker acted unequally and refused to treat a patient from another culture
Fear of negative consequences	Unfair, undignified acting towards a patient; a coworker handling the patient roughly
Fear of losing the job	A coworker (another nurse) acted unprofessionally in front of a patient, rudely pointing out a mistake which was not relevant or causing harm to the patient
Feeling powerless	A potentially self-harming, depressed patient did not get a place in the hospital ward despite the nurse trying their best, due to differing views between the nurse and the physician. The patient remained at home. In the end, the patient committed suicide
	A coworker handled the patient roughly and without dignity
Contextual	
The situation was over very quickly	A coworker used racist language about a patient, who also heard it
The situation was not severe for the patient (it was not worth taking the risk)	Violence of patient’s right to privacy
Another professional is not listening or does not seem to care	A physician was disrespectful towards patients and nurses
Organisational	
Hierarchy and a feeling that a nurse cannot confront a physician	A physician knowingly embarrasses a nurse in front of the patient
	Patients’ right to knowledge about their illness/condition is not actualised, because another professional does not want to tell difficult news
Feeling left alone in the organisation, no one cares about their opinion	A patient’s right to good care is not actualising, because a colleague isn’t doing their job

Individual justifications for not acting morally courageously were related to the nurses’ feelings and fears. For example, in a new workplace, a coworker spoke rudely about a small, non-hazardous mistake (that the nurse had done), in front of a patient. The justification for not acting morally courageously was the fear of negative consequences. ‘… I didn’t have the courage to say anything cos I was afraid that my work contract wouldn’t continue’ (Participant 8).

The contextual justifications were related to the severity, timing and other attributes of the situation. For example, a coworker used racist language about a patient, who heard it. The situation happened quickly, and it came as a surprise to the nurse, who did not act morally courageously. ‘… when the coworker said [the n-word] … so it just somehow came as a surprise for me, the situation that I didn’t even realise at the moment… that… what was really happening…’ (Participant 11).

The organisational justifications were related to other professionals and organisational matters, such as hierarchy. For example, in one case, the patient’s right to knowledge about their illness was not actualised, because a physician did not want to tell the difficult news. The justification for not acting morally courageously was a hierarchical decision and a notion that a young nurse could not confront physicians. ‘And then first you’re just that ok… the physicians are so experienced … and I assume they know what they are doing’ (Participant 3)

## Discussion

This study provides new knowledge on ethical conflicts where nurses need moral courage, the bases, and perspectives of justifications for whether to act morally courageously, and the morally courageous acts they perform. The starting point of the study was that for moral courage to manifest, there has to be an ethical conflict and a personal risk for the acting nurse.^1,2^ The nurses provided rich and detailed information about their experiences in their narratives. They expressed how they thoughtfully and deeply pondered the ethical conflicts and their justifications for whether to act morally courageously, and they tried to fulfil their ethical responsibilities.^36^ This illustrates the importance of moral courage for professional nursing ethics, as courage enhances acting according to what one considers to be right in an ethical conflict.^1^

The recounted ethical conflicts had emerged in situations with a patient or next of kin present and between professionals, corresponding with earlier research.^5,6^ Internal responsibility, professional ethics, and emotions were identified as bases of justifications. These did not exclude each other, but each nurse had one basis for justifications that was more important to them. The bases and perspectives are handled separately in the text to allow a broad understanding of the nurses’ justifications. One basis of justifications could be the most important for one nurse while varying perspectives of justifications were applied in different ethical conflicts. This illustrates the complexity of ethical conflicts and nurses’ decision-making when justifying morally courageous acts.^36^ The nurses mostly acted morally courageously in the ethical conflicts, which is promising and in line with earlier research.^9,11,13–15^ However, it is worth acknowledging that despite identifying the need for moral courage, the nurses sometimes decided to not act courageously. The justifications for not acting morally courageously add to the existing knowledge of inhibitors of moral courage.^37^ Additionally, the results correspond with Rest’s Four Psychological Components Determining Moral Behaviour, which include *moral sensitivity*: identifying the ethical conflict, *moral judgement*: pondering about the possible solution and choosing a justification for whether to act morally courageously; *moral motivation*: based on the justification, choosing a way of acting; and *moral character*: acting morally courageously based on the justification and decision.^38^ These four components illustrate moral courage as a fundamental part of an individual’s moral behaviour, essential to nurses regarding ethical competence and their actions in ethical conflicts.^2^

Due to the complex nature of ethical conflicts, one solution or justification does not fit all situations.^39^ Thus, nurses need the capability to justify their potentially morally courageous acts.^24,26,36^ Justifying acts in ethical conflicts is part of nurses’ value-based ethical decision-making, aiming to promote good and safe care.^36^ There are always other professionals, the patient, or their next of kin present in ethical conflicts, highlighting the importance of good, respectful communication in solving these conflicts.^12^ The ethical conflicts related to challenges in communication and collaboration in particular indicate that promoting respectful communication and collaboration between professionals can enhance nurses’ moral courage and the ethical conduct of professionals.^36,40^ This is at the core of nursing and nursing ethics: professionals working together for the good of the patient, based on the values of healthcare.^3,11,36,41^

It is notable that, even in similar areas of ethical conflicts, the justifications varied, illustrating the complexity of the ethical conflicts, nurses’ decision-making, and justifications.^6,21,26,36^ According to their narratives regarding their justifications for acting morally courageously, internal responsibility, professional ethics, and emotions were the bases of their justifications.^29^ The detailed narratives have been interpreted into these bases and perspectives to describe the justifications as clearly as possible, despite the complex world of nursing and nursing ethics.^42^

Nurses who felt internal responsibility as a basis of their justifications were aware of their values, and the justification for morally courageous acts often came from themselves. Their justifications indicate that they were committed to and willing to advocate for the patients.^1^ This highlights the crucial role of a morally courageous nurse as an individual who maintains an ethical quality of care.^1^ Professional ethics as a basis of justifications illustrated the nurses’ responsible conduct, following their professional ethical duties.^3^ Based on their acknowledgement of professional responsibilities in ethical conflicts, the nurses seemed to have good knowledge of professional ethics, which has been associated with moral courage among nurses.^2,9,10,15^ Those who based their justifications mainly on emotions wanted everyone to be well.^36^ Their pondering and weighing of the consequences for different persons involved in the conflict situation highlight their empathy and ethical decision-making skills.^36^ Although the nurses purposefully decided to follow the values of nursing,^3^ their justifications, confidence, and the boost to act came mainly from emotions, such as anger, that had arisen during the ethical conflict. This has previously been indicated in social psychology.^43^

The nurses who used all bases of justifications repeatedly acted morally courageously despite the risk of personal negative consequences. Thus, although the bases and perspectives of justifications varied, the nurses held the capability to justify and conduct their morally courageous acts. Often, justification came from an individual perspective, without help or support from others, illustrating the nurses’ capability to justify acting morally courageously. This perspective of justification is in line with earlier research, where moral courage has been described as having a strong professional identity, ethical sensitivity, and competence.^1,2,44^ However, sometimes the nurses decided to act morally courageously based on the conflict situation, indicating their skills in identifying an ethical conflict and acting according to what they considered to be right.^26,42^

As for the situations in which nurses did not act morally courageously, individual justifications were related to fears and feelings of powerlessness.^37^ These justifications sometimes prevented a nurse from acting morally courageously even when the situation was severe for the patient. The nurses could be afraid of possible negative reactions of other people.^37^ Contextual justifications for not acting morally courageously were related to the severity or the timing of the situation, hierarchy, and nurses’ feeling that they had been left alone. Situation severity as a justification can indicate that, in non-severe situations, nurses might feel that acting morally courageously would not be worth taking a personal risk.^37^ Regarding the justifications of hierarchy and nurses feeling left alone, it is relevant to acknowledge nurses’ right to a safe and positive work environment.^24,45^

To overcome hierarchical challenges and to promote moral courage among nurses, other professionals should show respect to nurses as professionals who can make moral decisions and act individually.^36,37^ Additionally, organisations should ensure strong and clear values to support nurses’ decision-making and actions in ethical conflicts.^11,46^ Furthermore, organisations can provide sufficient information, resources, and clear processes regarding ethical conflicts to support nurses who doubt their capabilities to act morally courageously or who cannot overcome their fears of negative consequences.^47^ This support can promote morally courageous actions among nurses despite the personal risks. With moral courage, it is possible to build moral resilience to continue to act courageously.^48^

### Strengths and limitations

The strengths and limitations of this study are related to sampling, data collection, data analysis, and reporting. The chosen online discussion forum, hoitajat.net, can be considered a strength because, although not all nurses use it, it is publicly accessible. Also, as a large online community, it had the potential to reach nurses with experience of ethical conflicts and moral courage. Three of the authors (E.P., H.L-K, and R.S) have backgrounds in nursing, which facilitated an understanding of the contexts the participants described. Additionally, this gave a possibility to deeply discuss the ethical conflicts during the interviews. Moreover, following the analysis frame systematically, and discussing the analysis regularly with the research team, strengthened the credibility of the study.

According to Lieblich et al., the strengths and limitations of a narrative inquiry can be evaluated through the dimensions of *width*, *coherence*, *insightfulness*, and *parsimony*.^29^ As a strength, *the width* of the interviews, the interpretation of the results, and their presentation can be identified from the quotations illustrating the participants’ unique voices. Additionally, the holistic content approach^29^ and the rich data provide varying examples in the narratives. This, in turn, allows for forming a comprehensive understanding of the ethical conflicts, nurses’ justifications, and acts that were described. *Coherence* refers to parts of the results fitting together to form ‘*a complete and meaningful picture’*.^29^ Another strength is that, regarding the ethical conflicts, the justifications for whether to act morally courageously and the acts themselves were presented holistically. However, the complexity of the holistic illustration can be considered a limitation. Furthermore*,* as a strength, *coherence* in relation to existing theories can be identified,^29^ with these results corresponding to Rest’s Four Psychological Components Determining Moral Behaviour.^38^ *Insightfulness* can be identified from the broader meaning and *a greater comprehension*^29^ of the results, illustrating the decision-making based on, and from perspectives of the justifications. Finally, *the parsimony* was aimed for by using ‘a small number of concepts’.^29^ However, as a limitation, the complex process of ethical conflicts and the bases and perspectives of justifications posed challenges to reaching *parsimony*.^29^

### Implications for further research

Further research could focus on the attributes of morally courageous nurses and their capability to justify morally courageous acts. The correlation between moral courage and moral resilience could also be explored to develop tools for maintaining and strengthening nurses’ moral courage. Additionally, it would be justified to develop functioning processes for solving ethical conflicts in healthcare organisations to promote nurses’ moral courage and ethical conduct. Finally, further research could identify possible ways to organise debriefings and analyse their outcomes when nurses experience, or are at risk of developing, moral distress or moral injury. This could support them in maintaining moral courage and developing moral resilience.

Further research could employ descriptive and exploratory designs to more deeply and widely understand moral courage among nurses. Also, action research involving nurses and healthcare organisations could be implemented to confirm the suitability of the interventions developed for the organisations’ needs and wishes. Using these research designs could add to the literature on nurses’ moral courage, which would deepen understanding and promote moral courage among nurses.

## Conclusions

The identified bases and perspectives of the justifications the nurses described illustrate the complexity of decision-making for nurses in ethical conflicts. Although the decision-making process is complex and the justifications vary, nursing practice and research can implement these results to develop processes to promote nurses’ moral courage and to examine relationships between moral courage and other concepts, such as moral resilience. Promoting the importance of nurses’ moral courage through research can help nurses strengthen and maintain it in practice.

## Supplemental Material


Supplemental Material - Nurses’ justifications for morally courageous acts in ethical conflicts: A narrative inquiry
Supplemental Material for Nurses’ justifications for morally courageous acts in ethical conflicts: A narrative inquiry by Elina Pajakoski, Helena Leino-Kilpi, Minna Stolt, Anto Čartolovni, and Riitta Suhonen in Nursing Ethics.
