# Supplementary material for: Nurses’ justifications for morally courageous acts in ethical conflicts: A narrative inquiry
Source: Nurs Ethics. 2024 Sep 26;32(3):752–66. doi: 10.1177/09697330241284357 (PMC11993820; doi:10.1177/09697330241284357)
Supplement: Supplemental Material - Nurses’ justifications for morally courageous acts in ethical conflicts: A narrative inquiry [file sj-pdf-1-nej-10.1177_09697330241284357.pdf]

Supplementary Table 1. Data analysis with the holistic content analysis

Phase		Act
**1**	Read	Reading each narrative several times, identifying meaningful contents according to the study objective
**2**	Identify (part I)	Individual ethical conflict<br>Justification for MC Act or no MC act<br>MC act
**3**	Identify (part II)	Identifying the areas of ethical conflicts, justifications and morally courageous acts.
**4**	Repeat	Phases 1 to 4 with each participant's narrative
**5**	Identify (part III)	Bases of justifications.
**6**	Form<br>forming tables	Forming perspectives of justifications for acting morally courageously
**7**	Identify (part IV)	Identifying the wholeness of:<br>- the ethical conflict<br>- justification for MC Act<br>according to each basis of justification
**8**	Identify (part IV)<br>and describe.	All nurses' justifications and their perspectives for not acting morally courageously.

MC = morally courageous
